# Metagenomic Geolocation Prediction Using an Adaptive Ensemble Classifier

**DOI:** 10.3389/fgene.2021.642282

**Published:** 2021-04-20

**Authors:** Samuel Anyaso-Samuel, Archie Sachdeva, Subharup Guha, Somnath Datta

**Affiliations:** Department of Biostatistics, University of Florida, Gainesville, FL, United States

**Keywords:** metagenomics, machine learning, ensemble classifier, microbiome, geolocation

## Abstract

Microbiome samples harvested from urban environments can be informative in predicting the geographic location of unknown samples. The idea that different cities may have geographically disparate microbial signatures can be utilized to predict the geographical location based on city-specific microbiome samples. We implemented this idea first; by utilizing standard bioinformatics procedures to pre-process the raw metagenomics samples provided by the CAMDA organizers. We trained several component classifiers and a robust ensemble classifier with data generated from taxonomy-dependent and taxonomy-free approaches. Also, we implemented class weighting and an optimal oversampling technique to overcome the class imbalance in the primary data. In each instance, we observed that the component classifiers performed differently, whereas the ensemble classifier consistently yielded optimal performance. Finally, we predicted the source cities of mystery samples provided by the organizers. Our results highlight the unreliability of restricting the classification of metagenomic samples to source origins to a single classification algorithm. By combining several component classifiers via the ensemble approach, we obtained classification results that were as good as the best-performing component classifier.

## 1. Introduction

The latest advancements in bioinformatics along with next-generation sequencing technologies have made metagenomic analysis affordable and approachable. Data from metagenomic sequencing technologies empower accurate estimation of the abundance of microbial communities in samples from different locations and environments. For a few years, the MetaSUB consortium has been collecting metagenomic samples from multiple cities around the globe with the aim of improved understanding of city microbes. One of the objectives of the data provided is to identify the source origin of metagenomic samples. Some previous studies have successfully mined the gut microbiome for extracting information related to the geolocation of the microbiome samples (Suzuki and Worobey, 2014; Clarke et al., 2017; Xia et al., 2019). For instance, the abundance of gut microbes such as Firmicutes and Bacteroides is associated with the samples collected from varying latitudes (Suzuki and Worobey, 2014). Likewise, microbiome samples collected from urban environments can be a potential source of information for geolocation predictions. Microbiome data analyzed by several teams that participated in previous critical assessment of massive data analysis (CAMDA) challenge corroborate this idea. Participants who have worked on this challenge in the past take different routes to analyze the data. For instance, Harris et al. (2019) use both assembly-based and read-based taxonomic profiling along with machine learning algorithms to predict source locations. They reported that the random forest gave the most promising prediction results. Ryan (2019) employs Kaiju for the taxonomic profiling of sequencing reads and utilized *t*-distributed stochastic neighbor embedding (*t*-SNE) for dimension reduction. This was followed by classification to city labels using the random forest. Zhu et al. (2019) emphasize the use of functional profiling of microbiome data over taxonomic profiling and applied support vector machines (SVM) to predict geolocation of unknown samples. While Kawulok et al. (2019) utilized a *k*-mer based approach to design fingerprints for the identification of source origins of metagenomics samples. In addition to predicting the source origin of microbiome samples, this field of research may also be advantageous from the public health point of view. Anti-microbial resistance is a valid threat to the treatment of infectious diseases. These studies can potentially play a significant role to gauge the prevalence of disease-causing microorganisms in our environment (Allen et al., 2010).

Issues encountered while analyzing metagenomic data are high dimensionality, class imbalance, and selection of the best classifier. In this study, we build a competent classification model to predict the geographic location of a metagenomic sample, along with addressing the aforementioned issues. Several standard classifiers have been employed for the prediction of the origin of a given microbial sample. The application of these individual classification algorithms yields a varying degree of classification accuracy, and the performance of such classifiers is also dependent on the structure of the available data. Rather than employing several individual classifiers, we adopt the adaptive ensemble classification algorithm proposed by Datta et al. (2010). The ensemble classifier, which is constructed by bagging and rank aggregation, comprises a set of standard classification algorithms where such individual algorithms are combined flexibly to yield classification performance at least, as good as the best classification algorithm in the ensemble. Besides, the modeling techniques presented in this study suggested that the ensemble classifier could be useful in other classification problems.

## 2. Methods

### 2.1. Data

The primary dataset provided by the organizers of the CAMDA challenge consists of (1) paired-end whole genome shotgun (WGS) metagenomics data of 1,065 samples from 23 cities across 17 countries and (2) metadata comprising of the biome, weather, and location information for these cities.

Furthermore, WGS data for 121 “mystery” samples from 10 different cities were subsequently provided by the organizers. Among the 121 “mystery” samples, 56 samples originated from cities that were not represented in the primary dataset. Cities in the mystery data which were not represented in the primary data include *Bogota, Krakow, Marseille, Naples*, and *Vienna*. The primary dataset corresponds to the “training set,” while the mystery samples make up the “test set” for validating the classification models built during the analysis phase of the study. Table 1 shows the information on the samples obtained from two collections (CSD16 and CSD17) for both the primary data and the mystery data.

**Table 1:** Number of samples from the primary data and the mystery data.

**Primary data**					**Mystery data**	
**Location code**	**Location**	**Country**	**# Samples**	**Avg. # of reads**	**Location**	**# Samples**
ARN	Stockholm	Sweden	50	1,621,983	Bogota	12
BCN	Barcelona	Spain	38	2,763,249	Hong Kong	15
BER	Berlin	Germany	41	6,095,554	Krakow	15
DEN	Denver	USA	45	2,293,732	Kyiv	11
DOH	Doha	Qatar	65	2,400,540	Marseille	10
FAI	Fairbanks	USA	48	6,860,242	Naples	9
HKG	Hong Kong	China	49	3,066,755	Taipei	11
ICN	Seoul	South Korea	50	3,053,297	Tokyo	14
IEV	Kyiv	Ukraine	49	2,179,260	Vienna	10
ILR	Ilorin	Nigeria	97	10,660,493	Zurich	14
KUL	Kuala Lumpur	Malaysia	30	2,310,143		
LCY	London	England	37	2,477,320		
LIS	Lisbon	Portugal	19	2,864,004		
NYC	New York City	USA	99	3,170,947		
OFF	Offa	Nigeria	26	22,772,079		
SAO	São Paulo	Brazil	29	1,989,278		
SCL	Santiago	Chile	26	10,399,795		
SDJ	Sendai	Japan	32	1,571,323		
SFO	San Francisco	USA	29	1,471,680		
SGP	Singapore	Singapore	48	2,761,780		
TPE	Taipei	China	50	2,755,260		
TYO	Tokyo	Japan	75	1,996,146		
ZRH	Zurich	Switzerland	33	2,827,183		

*The samples were sampled from two collections (CSD16 & CSD17) and obtained by the MetaSUB consortium. Average number of reads was obtained after performing quality control and pre-processing*.

### 2.2. Bioinformatics Pipeline

To prepare WGS data for downstream machine learning analysis, we construct a standard pipeline to carry out necessary bioinformatics procedures. First, we obtain the raw paired-end reads from the host server, and then, we perform some quality control and filtering to obtain good quality reads. After the quality control, we perform metagenomic taxonomic profiling of the sequencing reads. At the termination of the bioinformatics procedures, the final output is a relative abundance table. All the bioinformatics procedures carried out in this phase of our analysis were performed using the University of Florida HiperGator supercomputer.

The first procedure in the bioinformatics procedure entails the quality assessment of the WGS data using FastQC (Andrews, 2010) and MultiQC (Ewels et al., 2016). For each paired-end WGS data, FastQC provides simple quality control checks while we use MultiQC to aggregate the results from the FastQC reports into a single report for each city. After careful inspection of the city aggregated quality reports, we notice a good proportion of low-quality bases and adapter sequences in the WGS data, hence, we proceed to carry out quality filtering and trimming. Ideally, we clean up the data as well as reduce the size of the data to make the downstream analysis much convenient. We employed KneadData (McIver et al., 2017) for quality trimming and filtering, removal of adapter sequences, and discarding human contaminated reads. KneadData provides a wrapper script for some pre-processing tools to carry out quality control. First, we use kneadData to invoke Trimmomatic (Bolger et al., 2014) for trimming off reads or parts of reads that have low-quality scores, as well as removing adapter sequences. We define the parameters of the Trimmomatic program to ensure that adapter clipping is carried out before trimming, reads with a minimum length of 60 are retained, and also define a sliding window that cuts a read once the mean quality in a window size of 4 falls below a Phred score of 30.

Bowtie (Langmead and Salzberg, 2012) is subsequently employed to index the human reference genome, and reads that align to this reference genome are discarded. The reference genome was custom downloaded using the KneadData software version 0.7.4. Upon reassessment of the trimmed and filtered WGS data, we observe better quality and reduction in the file size.

After the standard bioinformatics pre-processing of the raw WGS reads, we proceed to taxonomic profiling. Here, we utilize the kraken2-bracken system (Lu et al., 2017; Wood et al., 2019) for the taxonomic profiling. In particular, we utilize kraken2 for the assignment of sequence reads to taxonomic labels and bracken for estimating taxa abundance. Kraken examines the *k*-mers in the given sequence reads and assigns taxonomic labels to the reads based on the similarity of the information in the *k*-mers in the sequence reads to the *k*-mer content of a reference genome database. The database maps *k*-mers to the lowest common ancestor (LCA) of all genomes known to contain a given *k*-mer. The LCA approach adopted by the Kraken system usually results in the underestimation of the number of reads which are directly classified at lower taxonomic levels (Lu et al., 2017). To overcome this problem, bracken is preferred for the estimation of relative abundance. Bracken (Lu et al., 2017) uses a Bayesian algorithm and the classification results obtained from the Kraken for the abundance estimation.

In application, we utilized a standard reference database comprised of reference sequences from archaea, bacteria, viruses, and the human genome to perform the taxonomic assignment. A bracken database file was generated with a default *k*-mer length of 35, and 150 base pair-reads. For each pre-processed pair-end sample, kraken2 classification reports were generated, and these reports were passed into the bracken program for estimation of species relative abundance. Finally, a custom bracken script was used to combine the taxonomic profile of each metagenomic sample into a large relative abundance table.

**Figure 1:**
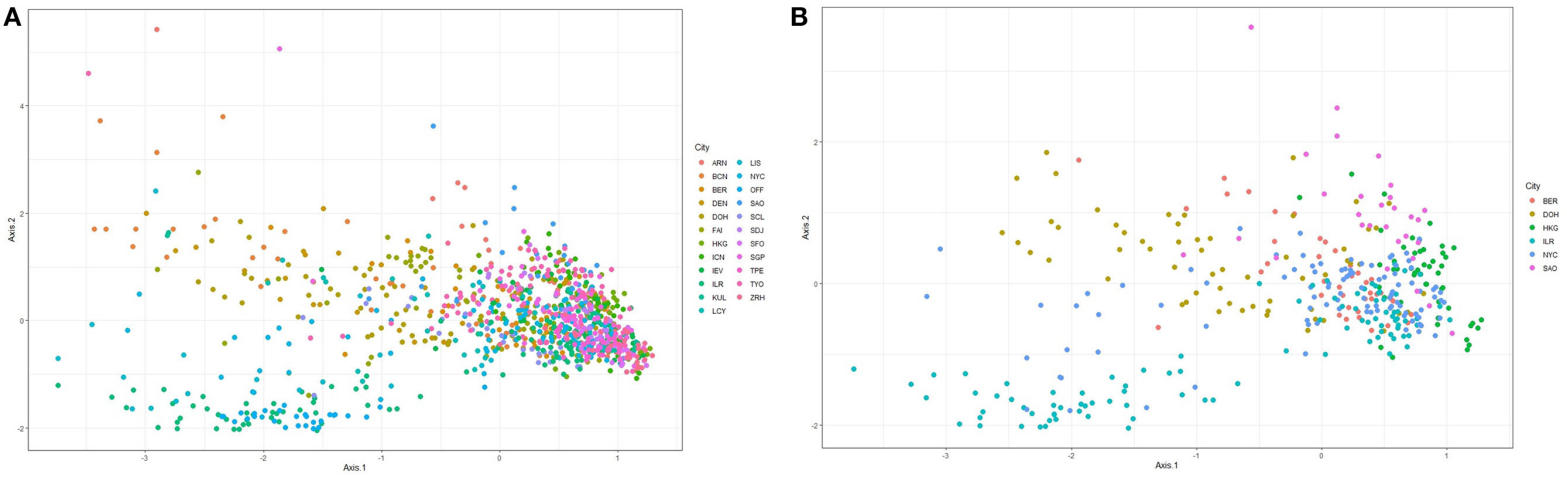
Ordination plots based on principal coordinate analysis with a unifrac distance for the relative abundance matrix consisting of **(A)** all cities in the primary data, and **(B)** at least one city from each continent represented in the primary data.

Figure 1 shows ordination plots for the resulting species abundance obtained from the Kraken2-Braken system. The ordination analysis is based on the method of principal coordinate analysis with a Unifrac distance (Lozupone and Knight, 2005). The left panel of Figure 1 shows the ordination plot for all 23 cities in the primary data set. For this plot, notice that the cities are not well-separated, a good portion of the samples overlap each other. While the right panel of Figure 1 shows the ordination plot for six unique cities with at least a city drawn from each continent represented in the primary data. Given the apparent overlap of the samples, it is an interesting pursuit to build robust classifiers that will learn to separate the cities using information from taxonomic abundance.

### 2.3. Pre-processing

At the termination of the bioinformatics pipeline, a species abundance table with 6,152 taxa for the 1,047 samples in the primary data is obtained from the Kraken2-Braken taxonomic profiling system. We conducted additional downstream pre-processing to identify and discard the least prevalent taxa in the abundance data. We removed taxa that were present in fewer than 1% of all samples, and further removed taxa with fewer than ten reads. Following these pre-processing steps, we re-normalize the data, and we obtain a species-level relative abundance table with 4,770 taxa.

### 2.4. Feature Engineering

After the initial preprocessing of the species-level abundance table, we get a data matrix **X** = ((*X*_*ij*_)), of dimension 1, 047 × 4, 770 where the rows represent samples from different cities, and the columns represent taxa. Here, the number of features is greater than the number of samples. It is usually desirable to reduce the feature space and thereby get a set of informative features that explain the variation in the samples. Several techniques exist for performing feature selection. For example, relevant features can be selected based on the *k* top-ranking importance scores returned by a fitted random forests model. To maximize classification accuracy, *k* can be chosen from a set of a predetermined number of features via cross-validation. The final classification model can then be trained with this set of *k* features.

For our analysis, we focus on the hierarchical feature engineering (HFE) method (Oudah and Henschel, 2018). In a nutshell, this technique uses correlation and taxonomy information in the abundance table as a heuristic to exploit the hierarchical structure of a set of different microbial communities for determining which microbial taxa are informative. Based on a predictive performance comparison following the implementation of the HFE algorithm and other standard feature selection methods such as Fizzy (Ditzler et al., 2015) and MetAML (Pasolli et al., 2016) that do not take the hierarchical structure of microbiome data into account, the developers of the HFE showed that their technique outperforms these methods. Motivated by this finding, we adopt the HFE for feature selection in our analysis. After the application of the HFE algorithm, we obtain a reduced feature version of the abundance table with informative taxa comprising of 144 high-level aggregations of taxonomic features, on average.

### 2.5. Handling Class Imbalance

In the primary data, there are *C* = 23 class labels (sample origins). There is an over-representation of certain classes in the primary data. For instance, *Ilorin* and *New York City* have 98 and 96 samples, respectively, whereas there are only 19 samples from *Lisbon*. Fitting machine learning models with an apparent class imbalance in the training data is likely to make the models to be biased toward the majority classes. In such a scenario, the fitted classifier is likely to predict the label of a random sample to be a majority class. Learning from imbalanced data is a popular topic in machine learning research, and the literature is rife with several techniques for handling such a problem. Amongst these techniques include down-sampling, up-sampling, hybrid methods, and class weighting. Down-sampling involves randomly removing samples from the majority classes until class frequencies are roughly balanced, while up-sampling involves randomly replicating instances in the minority classes to achieve the same size as the majority class. In practice, the utilization of these sampling techniques usually comes at a cost. For instance, down-sampling the majority classes results in potential loss of relevant information. While oversampling the minority classes is likely to introduce more bias into the fitted model.

Hybrid methods such as SMOTE (and its variants), AdaSyn, DSRBF, ProWsyn, and many others oversamples the minority classes by creating synthetic samples from those classes. Kovács (2019) gives an extensive comparison of minority oversampling techniques when applied to a large number of imbalanced datasets. Results presented in Kovács (2019) show variation in the performance of the oversampling methods when applied to several datasets. They suggested that careful investigation should be carried out before applying any oversampling technique to the classification problem. For the analysis of the data in this study, we assessed the performance of a variety of oversampling techniques presented in Kovács (2019) that apply to multiclass classification problems. The evaluation metrics presented in section 2.7 were utilized to assess performance. Among the oversampling techniques assessed, we observed that no single technique dominates others in performance. Further, we observed that GaussNoise (the introduction of Gaussian noise for the generation of synthetic samples) (Lee, 1999), AdaSyn (adaptive synthetic sampling approach for imbalanced learning) (He et al., 2008), and ProWsyn (Proximity Weighted Synthetic Oversampling Technique for Imbalanced Data Set Learning) (Barua et al., 2013) all gave a good performance. Besides, GaussNoise required less computational time. Thus, we utilized this technique to avoid increasing the complexity of the analytical process.

Moreover, class weights can be used to train several classifiers. In this sense, the algorithm puts more weight on the minority classes, thereby imposing a heavier cost when an error is made in the classification of labels from these classes. We define the class weights as $w_{c}=\frac{1}{n_{c}}$ for *c* = 1, …, *C* where *n*_*c*_ the number of samples from city *c*.

In a general sense, the implementation of these techniques does not improve the overall fit of the model, and they do not increase the information in the training data (Chen et al., 2004). However, these methods are employed to put more weight on the errors made in the minority classes. Consequently, they are designed to improve the prediction accuracy of the minority classes. In practice, these techniques are applied only to the training set and should be applied after the implementation of any resampling method (cross-validation (CV) or bootstrapping) used to evaluate model performance. The application of these techniques before train-test splitting or CV splitting will cause the trained model to give an overly optimistic estimate of model performance since the test set is likely to be familiar to the model.

### 2.6. Ensemble Classifier

In recent years, participants working on the metagenomic geolocation challenge have used a variety of popular classification algorithms. Support vector machines (SVM), random forests, and neural networks have been widely used for the prediction of the origin of a given metagenomic sample. For instance, Casimiro-Soriguer et al. (2019) used decision trees for the classification of functional profiles created from metagenomics data. The random forest classifier was used by Ryan (2019), Harris et al. (2019), and Walker and Datta (2019), while Zhu et al. (2019), and Walker and Datta (2019) used the SVM classifier. In some cases, several dimension reduction and feature engineering techniques have been applied in conjunction with the classification algorithms being utilized.

In principle, no single classification algorithm performs optimally on all types of data. In particular, the performance of the classification algorithm is likely to depend on the techniques used during the bioinformatics preprocessing and taxonomic profiling of the metagenomics data. The application of these individual techniques is likely to yield variable competing results, and notably, the classification accuracy from these methods is likely to depend on the structure of the available data. A similar observation was noted while discussing the results of a classification competition based on proteomics data (Hand, 2008).

In this study, we adopt a method for combining a variety of classification algorithms in addition to dimension reduction techniques (if necessary) rather than employing stand-alone classification algorithms for metagenomics classification problems. Datta et al. (2010) developed an adaptive ensemble classifier constructed by bagging and rank aggregation that yields near-optimal classification performance for different data structures. The ensemble classifier comprises a set of standard classification algorithms where such individual algorithms are combined flexibly to yield results that are better or as good as the best classification algorithm in the list of algorithms that make up the ensemble. In principle, the ensemble classifier adaptively adjusts its performance depending on the data being analyzed, and it reaches the performance of the best-performing individual classifier without explicitly knowing such classifier.

Next, we outline the steps presented by Datta et al. (2010) for the construction of the ensemble classifier. Consider the normalized relative abundance matrix of *n* samples and *p* taxa, **X**_*n*x*p*_ and the *n*-vector of classification labels, **y**. First, the data is partitioned into training and test sets. Set *N*, the number of independent bootstrap samples to draw from the training set. Also, select *M* candidate classification algorithms and *K* performance measures. Then the following iterative steps are performed for *j* = 1, 2, ..., *N*

1. Obtain the *j*th bootstrap sample. The bootstrap sample is obtained by simple random sampling with replacement and the sampling is performed such that all classes in the primary data are represented in the bootstrap sample. Samples that are not included in the bootstrap sample are called out-of-bag (OOB) samples.
2. Train all *M* classification models on the *j*th bootstrap sample.
3. Using the trained *M* classification models predict the class labels for the OOB set.
4. Based on the true values of the OOB set, and the predicted labels, compute the *K* performance measures.
5. Perform weighted rank aggregation: the performance measures used in step (iv) ranks the classifiers according to their performance under such measure, thereby producing *K* ordered lists, *L*_1_, *L*_2_, ..., *L*_*K*_, each of size *M*. Using weighted rank aggregation, the ordered lists are aggregated to determine the best single performing classifier denoted as $A_{\left(1\right)}^{j}$.

The ensemble is a set of $\left\{A_{\left(1\right)}^{1},...,A_{\left(1\right)}^{j},...,A_{\left(1\right)}^{N}\right\}$ classifiers.

To avoid overfitting the classifiers, the performance of each classifier is evaluated based on their prediction of the OOB samples. This approach is similar to the cross-validation procedure, and the misclassification error from the OOB samples approximates the testing set error.

Given a new sample **x**_1x*p*_, prediction using the ensemble classifier is based on the following procedures.

1. Each classifier, $A_{\left(1\right)}^{1},...,A_{\left(1\right)}^{N}$, in the ensemble is used the to predict the class label of **x**_1x*p*_. Let *ŷ*_1_, ..., *ŷ*_*N*_ denote the class predictions from the *N* models in the ensemble.
2. The final prediction is obtained by majority voting, that is, the most frequent label among the *N* predicted classes.

### 2.7. Analysis

For the analysis of the species abundance data obtained after bioinformatics pre-processing of the WGS data, we performed three distinct analyses for both the abundance table with a complete feature space and the table with a reduced feature space. We aimed to classify the species abundances of each metagenomic sample to a source city that is present in the primary data set. Our analyses focused on training standard classification algorithms, classification algorithms with class weights, and implementing an optimal over-sampling technique when training the classifiers. The implementation of the last two techniques was to handle the problem of class imbalance. Here, the candidate classifiers considered for the analyses include the random forest (RF) (Breiman, 2001), support vector machines (SVM) (Cortes and Vapnik, 1995) with a radial basis kernel, recursive partitioning (RPart) (Breiman et al., 1984), extreme gradient boosting (XGBoost) (Chen and Guestrin, 2016), and multilayer perceptron (MLP). These classifiers, in particular, RF and SVM have been used for metagenomics-based classification problems. However, we observed that the XGBoost classifier has not been frequently utilized. Also, we trained modifications of some of the mentioned classifiers, particularly we use principal component terms to train the RF classifier (denoted as PCA+RF) and partial least squares to train the RF, RPart, and XGBoost classifiers (denoted as PLS+RF, PLS+RPart, PLS+XGB).

Furthermore, for each analytical method presented, we constructed the ensemble classifier discussed in section 2.6 which comprises the chosen candidate classifiers. Note, the ensemble classifier may not be restricted to only distinct individual classifiers. Classifiers with different tuning parameter combinations can also be considered as candidate classifiers. Moreover, hyperparameters of the candidate classifiers can be tuned to attain optimal performance. However, tuning hyperparameters will increase the time it takes to construct the ensemble classifier.

A significant property of the ensemble classifier is its ability to simultaneously optimize classification results based on pre-specified model evaluation measures through weighted rank aggregation (Pihur et al., 2007) (see section 2.6). We chose to use Cohen's Kappa statistic (Cohen, 1960), multiclass geometric mean (denoted as G-mean) (Sun et al., 2006), and multiclass AUC (denoted as MAUC) (Hand and Till, 2001) as measures for performing weighted rank aggregation. The G-mean is the geometric mean of recall values for all classes, while the MAUC is the average AUC for all pairs of classes. These performance measures are defined as follows

$$\begin{array}{l} \;\kappa =\frac{P_{0}-P_{E}}{1-P_{E}},\\ \text{G-mean}={\left(\overset{K}{\underset{i=1}{\prod }}{\text{Recall}}_{i}\right)}^{\frac{1}{K}},\\ \text{ MAUC}=\frac{1}{K\left(K-1\right)}\overset{K}{\underset{i=1}{\sum }}\overset{K}{\underset{i\neq j}{\sum }}\text{AUC}\left(i,j\right), \end{array}$$

where *P*_0_ is the relative observed agreement among classifiers (i.e., the overall accuracy of the model), *P*_*E*_ is the probability that agreement is due to chance, *K* is the number of classes, and Recall_*i*_ is the recall for class *i*. For classification problems with imbalance data, these measures are usually preferred over say, the classification accuracy. Since accuracy is only marginally impacted by rare classes in the data, the classification accuracy is often not an appropriate measure to employ for imbalance learning (Joshi et al., 2001).

## 3. Results

We present the results of the classification analysis in two sections. First, we describe results based on the primary data that consists of species abundance of metagenomic samples obtained from the 23 cities. Secondly, we present classification results from the analysis of the mystery samples. We also demonstrate the application of the ensemble classifier on the data generated from a taxonomy-free approach. This is described in detail in section 3.3.

### 3.1. Classification Results of the Primary Data

Species abundance data were obtained after taxonomic profiling with the kraken2-bracken system, the data were subjected to further downstream pre-processing as discussed in section 2.3, and the final abundance matrix contained 4,770 taxa. We refer to this data as the primary data with a complete feature space. Furthermore, the application of the HFE to the primary data resulted in about an 83% reduction of the feature space, on average. This section describes classification results for the analysis for both the primary data with a complete feature space and the data with a reduced feature space.

The classification results are based on a 10-fold split of the data into 80% training set and 20% test set. We ensured that at least three samples from each city were present in both training and test sets. The training sets were used to fit classification models while the test sets were utilized for validation. Consequently, the classification results presented here are based on the performance of the classifiers on the test sets. For the construction of the ensemble classifier, we set *N*, the number of bootstrap samples, to be 50. G-mean, Cohen's kappa statistic, and MAUC were all utilized for performing weighted rank aggregation, and for the overall evaluation of classification performance.

For the analysis of the primary data set with a complete feature space, the averaged performance measures for the set of candidate classifiers and the ensemble classifier are shown in Supplementary Table 1, a subset of this large table is presented in Table 2. The sub-tables of Table 2 shows the results for the standard classifiers, the classifiers trained with class weights, and the classifiers for which an over-sampling procedure is implemented, respectively. For the analysis of the data with a reduced feature space, Supplementary Table 2 shows the classification results. Again, we present a subset of the large table for the results on the classification of the HFE data in Table 3. Since the classification accuracy may be of interest to some readers, we have also supplied this information in the tables shown in the Supplementary Material. Also, the last columns of the sub-tables show the number of times each candidate classifier was the best performing local classifier in 500 instances (10 replicates each with 50 bootstrap samples).

**Table 2:** The mean performance measures for a set of candidate classifiers and the ensemble classifier.

	**Standard**			**Weighted**			**Over-sampling**		
**Classifier**	**Kappa**	**MAUC**	**Count**	**Kappa**	**MAUC**	**Count**	**Kappa**	**MAUC**	**Count**
Ensemble	0.89	0.93	–	0.87	0.92	–	0.87	0.93	–
MLP	0.84	0.91	125	0.80	0.89	159	0.80	0.90	118
PCA+RF	0.03	0.64	0	0.04	0.66	0	0.03	0.66	0
PLS+RF	0.88	0.92	239	0.87	0.92	235	0.86	0.92	199
PLS+RPART	0.55	0.74	0	0.51	0.76	0	0.49	0.75	0
PLS+XGB	0.83	0.89	0	0.81	0.89	1	0.80	0.88	1
RF	0.85	0.91	18	0.84	0.92	47	0.81	0.91	38
RPART	0.54	0.76	0	0.56	0.79	1	0.54	0.78	0
SVM	0.76	0.87	1	0.00	0.50	0	0.72	0.86	0
XGB	0.88	0.93	117	0.86	0.91	57	0.84	0.91	144

*Each classifier was trained on the primary data with a complete feature space. Three different scenarios are considered—the standard classifiers, classifiers trained with class weights, and classifiers trained with the implementation of an over-sampling procedure*.

**Table 3:** The mean performance measures for a set of candidate classifiers and the ensemble classifier.

	**Standard**			**Weighted**			**Over-sampling**		
**Classifier**	**Kappa**	**MAUC**	**Count**	**Kappa**	**MAUC**	**Count**	**Kappa**	**MAUC**	**Count**
Ensemble	0.85	0.92	-	0.86	0.92	–	0.82	0.91	–
MLP	0.73	0.86	8	0.71	0.86	9	0.69	0.84	5
PCA+RF	0.04	0.65	0	0.04	0.64	0	0.06	0.67	0
PLS+RF	0.77	0.86	4	0.75	0.86	9	0.71	0.85	5
PLS+RPART	0.36	0.68	0	0.31	0.71	0	0.30	0.71	0
PLS+XGB	0.70	0.82	2	0.70	0.84	2	0.67	0.83	0
RF	0.85	0.92	260	0.86	0.92	372	0.83	0.91	290
RPART	0.53	0.76	1	0.55	0.79	0	0.51	0.76	0
SVM	0.36	0.69	0	0.00	0.50	0	0.41	0.72	0
XGB	0.85	0.92	225	0.84	0.90	108	0.82	0.90	200

*Each classifier was trained on the primary data with a reduced feature space. Three different scenarios are considered—the standard classifiers, classifiers trained with class weights, and classifiers trained with the implementation of an over-sampling procedure*.

To assess the impact of the (HFE) dimension reduction technique applied in the analysis, we compare the classification results shown in Tables 2, 3. For most classifiers and each model fitting procedure, there is only little variation between the performance measures when the classifiers are trained on data with a complete feature space and with a reduced feature space. A superficial examination of both tables might suggest that classifiers trained with complete feature space give a slightly better overall performance. However, in terms of computational efficiency, the computational burden of training the classifiers is substantially reduced when they are trained on the data with reduced feature space.

Furthermore, to assess the impact of the methods used to handle the class imbalance problem, we compare the classification results across the standard models, the models with class weighting, and models for which an oversampling procedure is implemented. For the classifiers trained with class weights, the weights were computed as $w_{c}=\frac{1}{n_{c}}$, where *n*_*c*_ is the number of samples in class *c*. For the oversampling procedure, we employed the GaussNoise oversampling technique. For each classifier in either Table 2 or Table 3, we observe that there are only marginal differences among the three methods. The set of candidate classifiers that give good classification performance for one method is largely the same for the other methods. This result expounds on the point that the techniques used to address class imbalance do not improve the overall fit of the classification model.

**Figure 2:**
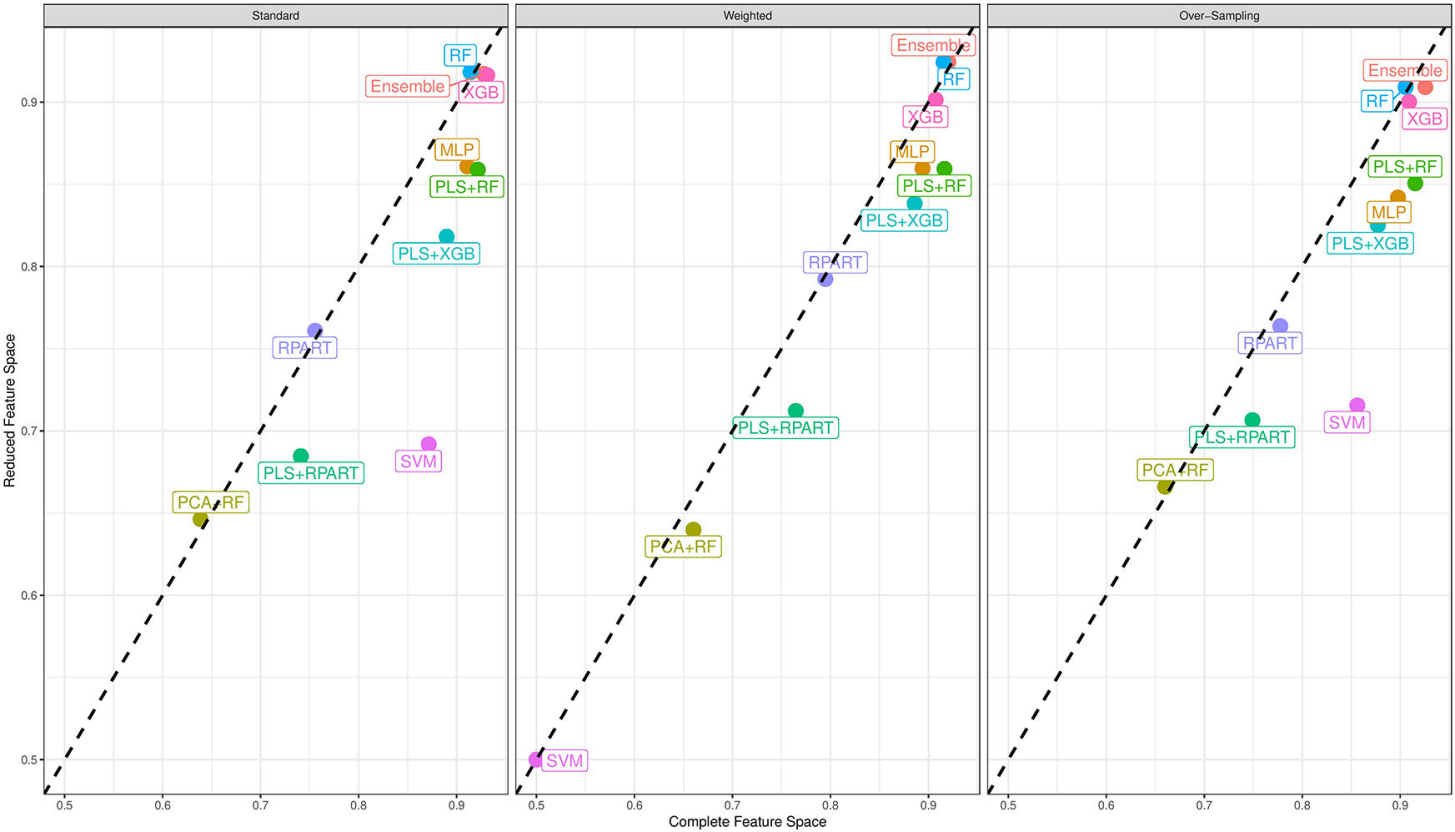
Mean multiclass AUC (MAUC) score for nine candidate classifiers, and an ensemble classifier comprising of the candidate classifiers. These classifiers are trained with the primary data with a complete feature space and the data with a reduced feature space.

Now, we turn our attention to the performance of each classification algorithm. Across the different analyses conducted and for the performance measures utilized, the ensemble classifier gives classification results as good as the best performing candidate classifier. Also, we observe the variation of classification performance among the candidate classifiers. The MLP, PLS+RF, RF, PLS+XGB, and XGB classifiers were mostly the best-performing candidate classifiers. Also, the RPart, PLS+RPart, and the SVM give moderate classification performance. For the analysis of the data with complete feature space, the classifiers trained with integrated PLS terms give very good classification performance. On the contrary, classifiers trained with integrated PCA terms were the worst performing classifiers. Figure 2 shows the mean MAUC scores for each classifier when trained and evaluated with both the data with complete feature space and the data with reduced feature space. From the evaluation of classification performance from this plot, most classifiers give better results when trained with the complete data than the reduced feature version.

Moreover, from the last column of the subtables of Tables 2, 3, we observe that the ensemble classifier is mostly dominated by the MLP, PLS+RF, RF, and XGB classifiers when these classifiers are trained with the data with complete feature space, while for the data with a reduced feature space, the ensemble classifier is mostly dominated by the RF and XGB classifiers. The effectiveness of the PLS terms incorporated for dimension reduction is substantially reduced for the analysis of the data with a reduced feature space, and the MLP classifier gives better performance for the analysis of the high-dimensional data. Both RF and XGB classifiers perform well in either setting.

**Figure 3:**
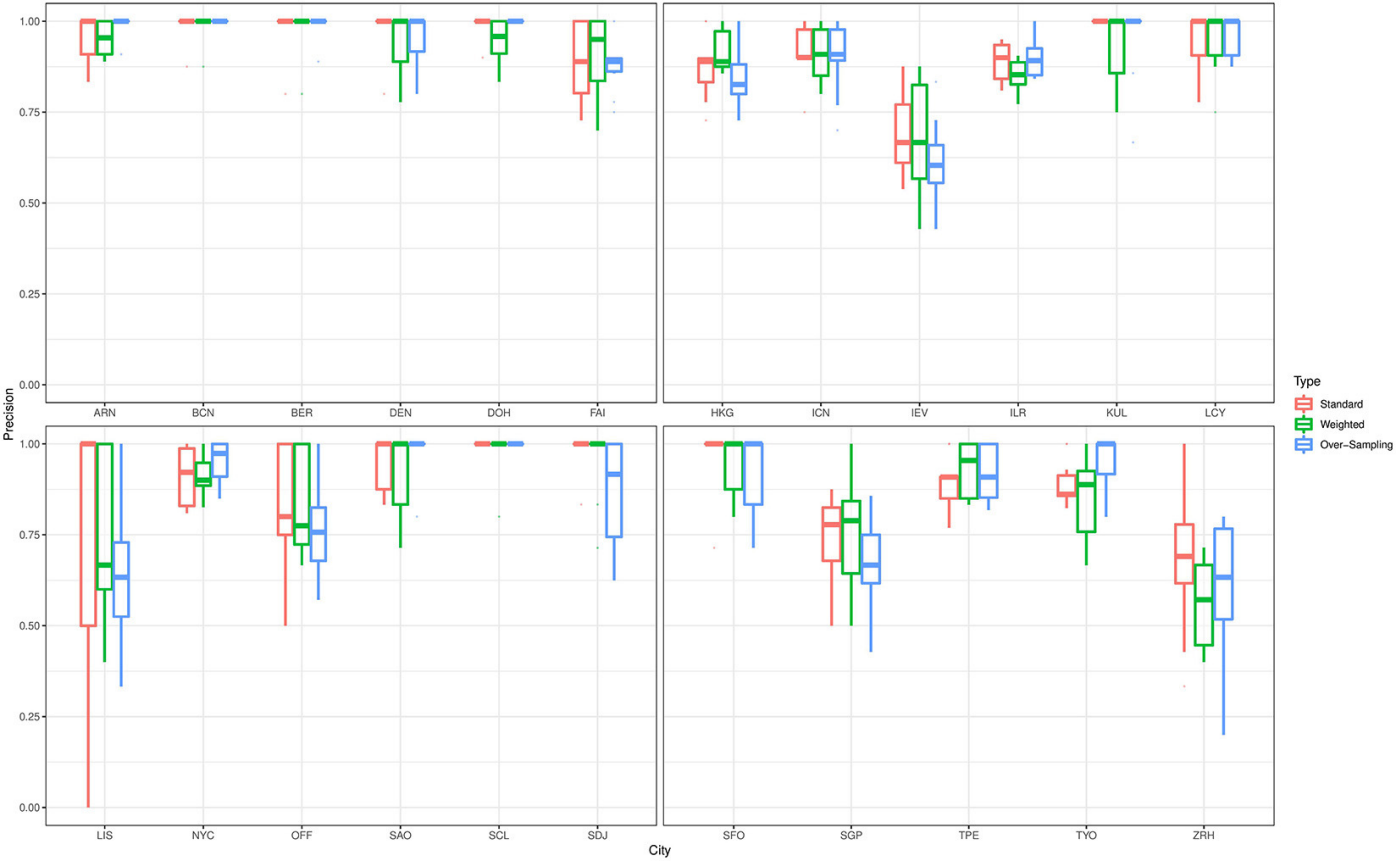
Precision values for all cities in the primary data with complete feature space. The results are based on predictions from an ensemble classifier for which the candidate classifiers were respectively trained using their standard version, and when class weighting and over-sampling procedures were implemented.

For the prediction of the source origins, the classifiers give a varying performance for predicting the cities in the primary data. To elaborate on this point, we describe the prediction performance of the ensemble classifier that was trained on data with complete feature space. Figure 3 shows boxplots for the precision values for all cities in the primary data that were based on predictions from the ensemble classifier. To train the classifier, we employed the standard technique as well as the class weighting and over-sampling procedures. Irrespective of the technique applied, the classifier gives excellent prediction results for *Stockholm, Barcelona, Berlin, Denver, Doha, Hong Kong, Seoul, Ilorin, Kuala Lumpur, London, New York City, São Paulo, Santiago, Sendai, San Francisco, Taipei*, and *Tokyo*. The average precision value for these cities was at least 85%. On the other hand, the classifier gave only moderate prediction result for *Kyiv, Lisbon, Singapore*, and *Zurich*. For the prediction *Lisbon*, which is a minority class in this study, the average precision value substantially increases from 66 to 73% when the class weighting procedure is employed as against the standard fit. However, we do not see the same effect when the over-sampling technique is applied. This empirical finding may be due to oversampling employed, further investigation will be done elsewhere to understand the impact of oversamplers in the analysis of microbiome data. Further, we observe the classifier does not perform well in distinguishing samples from *Kyiv* and *Zurich*, this pattern also persists for *Ilorin* and *Offa*. Geographically, *Ilorin* and *Offa* are two urban centers that are about 59.6 km apart in the same territory in Nigeria, whereas *Kyiv* and *Zurich* are European cities that are over 2,000 km apart. Also, we noticed a reverse misclassification between *Singapore* and *Kyiv*, these cities lie on different continents.

**Figure 4:**
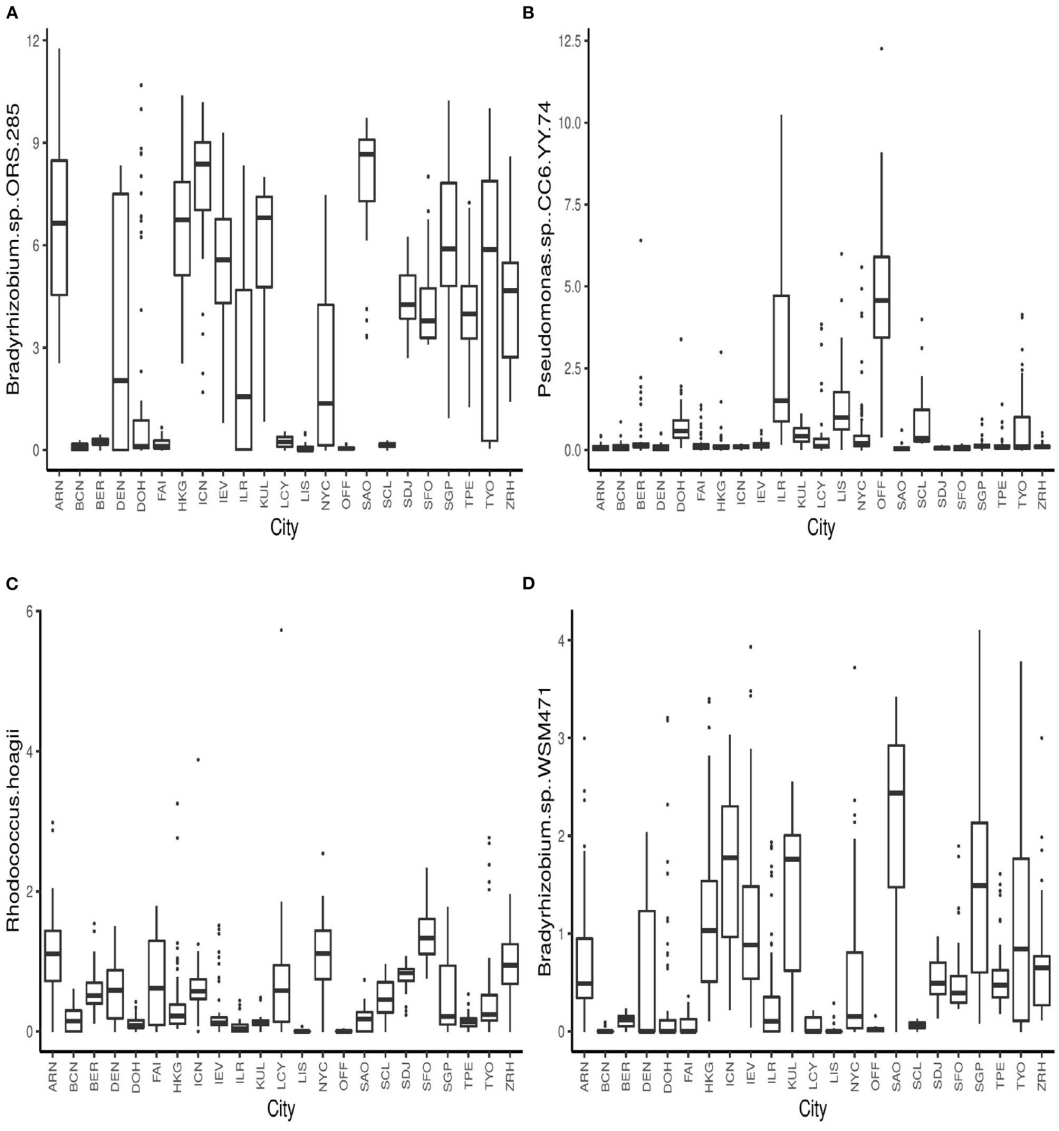
Boxplots of relative abundances of species **(A)** *Bradyrhizobium*..sp.ORS.285 **(B)** *Pseudomonas*..sp.CC6.YY.74, **(C)** *Rhodococcus*.hoagii, and **(D)** *Bradyrhizobium*.sp..WSM471 across all 23 cities.

**Figure 5:**
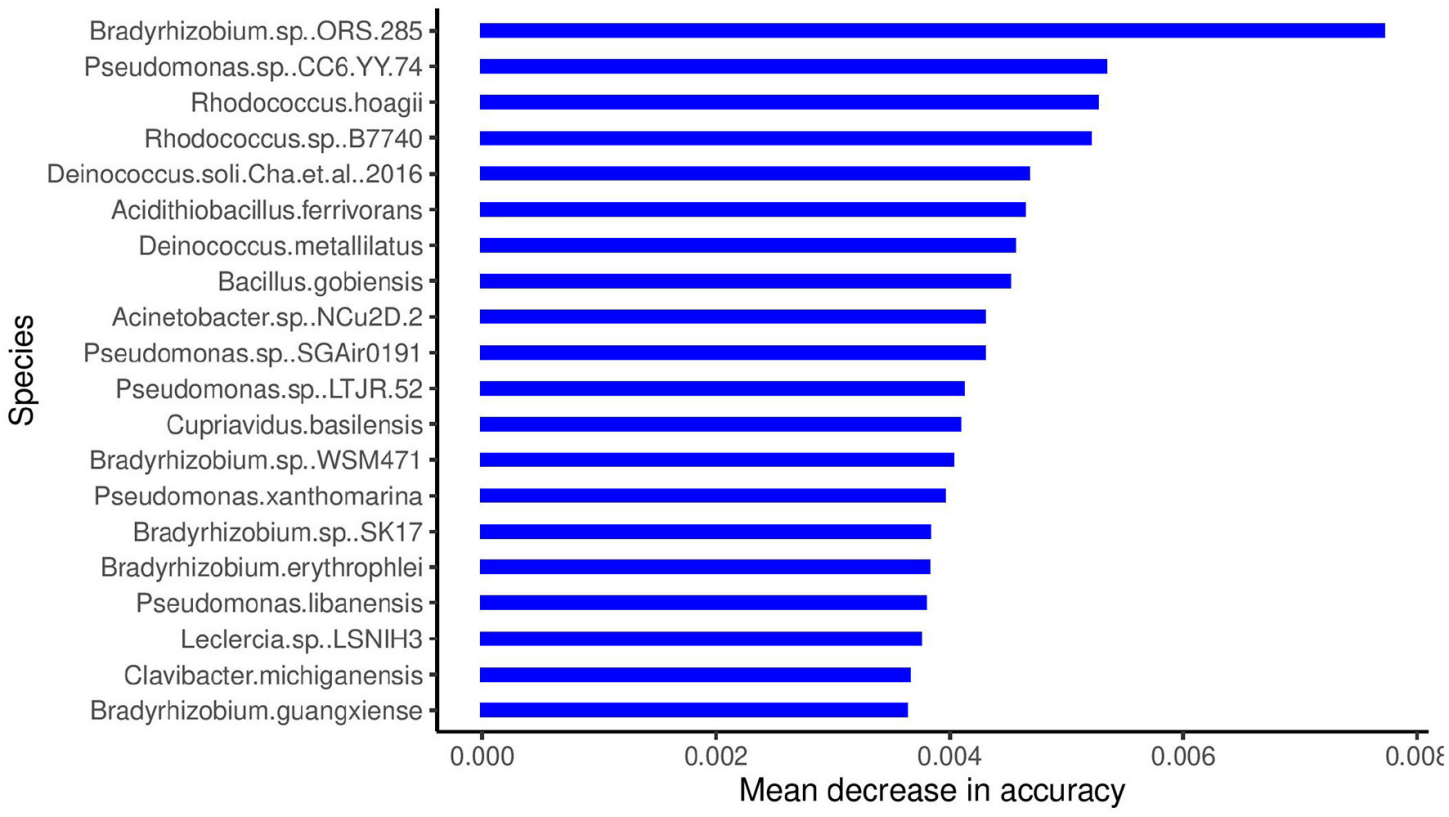
Species importance for RF classifier in the ensemble.

The boxplots in Figure 4 show some of the top microbial species that were found to be differentially abundant across various cities. Figure 5 shows the feature importance plot of the top 20 species from the RF classifier in the ensemble. Variable importance plot consists of many species belonging to genus *Bradyrhizobium* which is a soil bacteria and is also found in the roots and stems of plants (Giraud et al., 2013). It is interesting to note that species *Pseudomonas.sp.CC6.YY.74* is highly abundant in *Ilorin* and *Offa* that are geographically closer.

### 3.2. Analysis of Mystery Samples

In section 2.1, we described the mystery samples provided by the organizers of the challenge. For the bioinformatics and downstream pre-processing of the raw WGS data, we follow the same procedures discussed in sections 2.2 and 2.3, respectively.

From Table 1, there are 56 samples in the mystery data whose origins were not represented in the primary data. Since the ensemble classifier is a supervised classifier, it will not be able to be capable of predicting source cities that were not in the training data. For this reason, we restrict the analysis of the mystery data to samples for which their source cities were represented in the primary data. To predict these source cities, we use the entire primary data to train the ensemble classifier. For the construction of the ensemble classifier, the standard classifiers presented in section 2.7 were the base classifiers. Also, each classifier was trained with class weights on 50 bootstrap samples. Other elements of the ensemble classifier follow the input values presented in section 3.1.

The ensemble classifier reached an overall classification accuracy of 72% for the prediction of the subset of mystery samples. The best performing candidate classifiers were PLS+RF and XGB both of which also reached 72% accuracy, while the MLP and RF classifiers reached 68 and 57% accuracy respectively. Upon investigation of the classification results obtained from the ensemble classifier, we observed a high misclassification rate for the prediction of samples that originated from *Kyiv* and *Zurich*. Similar to findings presented in section 2.7, the ensemble classifier tends to misclassify the source origin of a sample from *Zurich* as being from *Kyiv*, and samples from *Kyiv* as *Singapore*.

As an alternative to the prediction of a mystery sample, the source city that minimizes the Kullback-Leibler (KL) distance between the mystery sample and the samples in the primary data is obtained as the prediction for the mystery sample. In this projection approach, for each mystery sample, we compute the KL distance between the mystery sample and all samples in the primary data. The city in the primary data that minimizes the KL distance is selected as the prediction of the source origin for the mystery sample. Following this approach, we obtained a classification accuracy of 52%. For each mystery sample, we also considered the source cities corresponding to the least three KL distances, we noticed that the true source origin falls in the set of the three cities 66% of the time.

### 3.3. Taxonomy-free Approach

The results discussed in section 3.1 were based on the analysis of the abundance data obtained after taxonomic profiling of the WGS data with the kraken2-bracken system. Typically, the taxonomic profiling step involves classification or mapping of sequence reads to their taxon of origin using a well-curated reference database of microbial genomes. For instance, kraken2 (Wood et al., 2019) utilizes the National Center for Biotechnology Information's (NCBI) RefSeq database for taxonomy classification. The software also gives users the choice of utilizing custom-built databases. Abundance estimation of each taxon in the metagenomic sample follows after the mapping step. In several cases, a good portion of the sequence reads remains unclassified after the taxonomic profiling step. A potential reason for the non-classification of sequence reads is that the taxa for some novel genetic materials in the metagenomic samples are not represented in the reference database. Therefore, a possible drawback of constructing microbial fingerprints with only information from taxonomic profiles is that unclassified DNA reads that may correspond to novel taxa are not considered during downstream analysis.

To overcome this drawback, several studies have suggested the construction of microbial fingerprints based on the primary modeling of the information contained in the sequence reads. For instance, (Kawulok et al., 2019) constructed a fingerprint that comprises a set of *k*-mers derived from metagenomics samples. In their approach, they studied the similarity between a sample and a reference *k*-mer database obtained at the reads level. Further, they showed that their method yields results that are as good as methods that rely on taxonomic/functional profiles of the metagenomics samples.

In this section, we describe the design of a microbial fingerprint by the direct modeling of DNA fragments obtained from metagenomics samples using an ensemble classifier. The DNA fragments are derived by breaking down the sequence reads into short sequences composed of *k* symbols (*k*-mers). For each sample obtained after the bioinformatics pre-processing step described in section 2.2, we utilize the KMC 3 (Kokot et al., 2017) software to obtain the frequency of unique *k*-mers. The KMC 3 software extracts *k*-mers with nucleotide alphabets (“A”, “C”, “G”, “T”), and it ignores *k*-mers containing “N” symbol (ambiguous nucleotides). We set *k* = 31, and we filtered out *k*-mers occurring less than 20 times (this threshold was set to 50 for samples gotten from *Offa* and *Ilorin*). For each city, we merged the *k*-mer frequency vectors generated for all samples. We observed a significant variation in the total number of unique *k*-mers obtained for the samples in each of the 23 cities in the primary set. For instance, there were a total of 6M, 47M, 268M, and 39M unique *k*-mers for the samples obtained from *Denver, Hong Kong, Offa*, and *Zurich*, respectively. We observed the samples in the mystery set generated a total of just 17M unique *k*-mers. Supplementary Table 3 shows the total number of unique *k*-mers corresponding to each of the 23 cities in the primary set and the mystery set.

After combining the *k*-mer frequency vectors from all available samples, we obtain a large sparse matrix with 923M unique *k*-mers. We performed some pre-processing on the matrix. First, we filter the data matrix to retain only *k*-mers that are present in at least 10% of the samples. A total of 298,644 *k*-mers were retained after filtering. Secondly, we obtain normalized counts for each *k*-mer corresponding to each sample.

To fit the ensemble classifier, we utilize the same settings discussed in sections 2.7 and 3.1. Using the normalized *k*-mer frequencies as feature vectors, we train the classifiers with class weights and without class weights. First, we describe the results for the analysis of the primary data. Table 4 shows the average performance measures for each candidate classifier and the ensemble classifier. For both the weighted and unweighted analysis, the RF and the XGB classifiers give the best classification performance among the candidate classifiers. From Table 4, we observe that the ensemble classifier trained with the *k*-mer frequency vectors gives a comparative performance as to when trained with taxonomic profiles. In contrast, there is greater diversity among the classifiers that make up the ensemble for predicting the abundance data, whereas the XGB and the RF (in particular) dominate the ensemble for the *k*-mer counts data. Also, we observe that the utilization of feature reduction terms (PLS and PCA) when training the classifiers based on the *k*-mer frequency counts does not yield better classification results.

**Table 4:** The mean performance measures for a set of candidate classifiers and the ensemble classifier based on the analysis of *k*-mer frequencies.

	**Standard**					**Weighted**				
**Classifier**	**Accuracy**	**G-Mean**	**Kappa**	**MAUC**	**Count**	**Accuracy**	**G-Mean**	**Kappa**	**MAUC**	**Count**
Ensemble	0.87	0.86	0.86	0.93	–	0.87	0.86	0.86	0.93	–
MLP	0.30	0.00	0.26	0.72	0	0.28	0.00	0.25	0.71	0
PCA+RF	0.09	0.00	0.03	0.67	0	0.12	0.00	0.07	0.67	0
PLS+AdaBoost	0.34	0.00	0.30	0.70	0	0.35	0.00	0.31	0.70	0
PLS+RF	0.38	0.00	0.35	0.71	0	0.40	0.00	0.37	0.70	0
PLS+RPART	0.31	0.07	0.27	0.71	0	0.26	0.05	0.23	0.71	0
PLS+XGB	0.37	0.00	0.33	0.70	0	0.39	0.00	0.35	0.70	0
RF	0.87	0.86	0.86	0.93	358	0.87	0.86	0.86	0.93	440
XGB	0.86	0.84	0.85	0.92	142	0.86	0.84	0.85	0.92	60

*Two different scenarios are considered—the standard classifiers and classifiers trained with class weights*.

For predicting the samples in the mystery set, we restrict the analysis to samples whose source origins were present in the primary data. Overall, the classification accuracy for the mystery set using this approach was much worse than the accuracy for the primary dataset and the taxonomy-dependent approach. For the *k*-mer approach, the ensemble classifier that comprises unweighted classifiers reached a classification accuracy of 35%, while the ensemble classifier based on weighted classifiers attains 33% accuracy. Some potential reasons could explain the discrepancy in the classification performance for samples in the primary set and the samples. First, with the diversity of samples in the mystery set (121 samples from 10 different cities), one would expect the total number of unique *k*-mers obtained from the mystery set to be greater than the total number of *k*-mers obtained from any of the 23 cities in the primary set. However, from Supplementary Table 3, we observe the total number of unique *k*-mers from the mystery set was lesser than the amount returned from 15 cities. Secondly, the discrepancy may be due to the batch effects for the primary and mystery data, and there may have been a difference in the sequencing procedures utilized for each batch. If there was a difference in the techniques used to generate the raw WGS data, the taxonomy-free approach appears not to be agnostic to the different techniques. Note, we implemented a uniform bioinformatics pipeline for the pre-processing of the raw WGS data. Lastly, when training the classifier with *k*-mer frequency vectors, it is difficult to know if the classification is driven by noise or pure biological relevance. For instance, we observed little or no changes in the classification performance when we discard *k*-mers that were not present in more than 1 to 30% of the total number of samples in the primary data. Besides, when feature reduction is implemented using *t*-SNE, PLS, or PCA, we observed the classifiers give poor results. In contrast, when we train the classifiers with an abundance matrix, classification is driven by taxonomy profiles that have biological meaningfulness.

We attempted yet another taxonomy-free approach called Simka (Benoit et al., 2016) to predict the source origins of the mystery samples. Simka computes the ecological distance between the samples based on their *k*-mer counts. It considers *.fastq* files as the input and processes the data in two major steps. The first step computes an *n* × *p k*-mer count matrix, where *n* is the number of samples and *p* is the number of distinct *k*-mers among all the samples. Based on the *k*-mer count matrix in the first step, it computes a *n* × *n* distance matrix. Table 1 of Benoit et al. (2016) describes the different ecological distance measures computed by Simka. We performed a naive distance-based method to predict the source cities for the mystery samples. Using the distance matrix obtained from Simka we computed the average distance of each mystery sample from the samples belonging to each of the 23 cities. For mystery sample *S*_*j*_, we obtained average distance ${\overset{-}{d}}_{j,1},\cdots \;,{\overset{-}{d}}_{j,23}$, where ${\overset{-}{d}}_{j,1}$ corresponds to the mean distance of mystery sample *S*_*j*_ from all samples in city 1 (e.g., ARN). The city label that corresponds to the minimum distance from mystery sample *S*_*j*_ is regarded as the predicted city label for that mystery sample. We utilized several different distance measures to implement the described method. The accuracy for the 18 distance measures that we used, varied from 0 to 28.57%. We observed that the qualitative distance measures i.e., based on presence-absence data had slightly higher accuracy than quantitative distance measures, i.e., based on abundance data. The 28.57% prediction accuracy for mystery samples was obtained by using qualitative *Kulczynski* distance measure. The average distance in the above method was computed using leave-one-out cross-validation to mitigate overfitting.

## 4. Discussion

In this study, we have presented several analytical approaches for the classification of abundance profiles from metagenomic samples to known source origins. We assessed the impact of dimension reduction, procedures for handling class imbalance, and using an ensemble classifier in downstream analysis. First, we observed that dimension reduction does not improve the classification performance of the models. There is only little variation in the prediction results obtained from the classification models trained with metagenomics data with a complete feature space and the data with reduced feature space. For large-scale studies with very high-dimensional data (that is, situations where the number of taxa obtained after taxonomic profiling is large), the analyst may prefer to adopt the analysis based on data with reduced feature space since such case will have a lesser computational burden.

To address the problem of class imbalance, we trained the classifiers with class weights, and we also adopted an oversampling technique. We observed that there was no substantial improvement in the overall classification performance when these methods were employed. In a microbiota study, Knights et al. (2011) utilized an oversampling approach to increase the number of samples in their case. They also reported a marginal difference in prediction performance when this approach is applied. In contrast, Harris et al. (2019) reported a considerable amount of increase in classification accuracy after an optimal oversampling technique was utilized for classifying metagenomics data to source cities. Oversampling and class weighting techniques are designed to improve the predictions made in rare classes. From our analyses, we observed that class weighting produced substantial improvement for the prediction of the minority class while oversampling did not have a major impact. In the future, we expect to investigate the impact of these methods in the analysis of microbiome data. Moreover, training classifiers with class weights are more computationally efficient than incorporating an oversampling procedure.

The ordination plots in Figure 1 depict that there is no clear separation between the microbiome sample from different cities. Thus, a major challenge in metagenomic geolocation data analysis is to identify a robust classification algorithm that can classify samples to their source cities with good prediction performance. To achieve this goal, we employed an ensemble classifier that comprised a set of several candidate classification algorithms for better prediction of the source origin of metagenomic samples. From the analyses presented in this study and other analyses presented in past CAMDA metagenomics forensics challenges, we observe that stand-alone classifiers are prone to give varying classification performance when different data structures are considered. The structure of the training data used to fit the classification models is likely to depend on the bioinformatics procedures employed for pre-processing of the raw sequence reads and the taxonomic profiling technique used for obtaining the abundance table. For this reason, it becomes unreliable to restrict the classification of abundance profiles of samples to source origins, to a single classifier.

Also, for any classification problem, it is natural for the analyst to consider a variety of classifiers. This usually depends on the objective of the scientific study under investigation. In practice, after carrying out standard pre-processing, the analyst initiates the classification analysis by evaluating the performance of simple models in classifying the abundance data to known class labels before experimenting with other advanced models. Based on user-specific performance metrics, the ensemble classifier offers the analyst the opportunity to evaluate the performance of different candidate classifiers on the chosen dataset in one shot. The ensemble classifier adapts to various bioinformatics procedures performed during pre-processing and taxonomic profiling of raw metagenomics data and will outperform or will adequately compete with any stand-alone classifier in its ensemble.

Moreover, before fitting the ensemble classifier, the analyst does not know the candidate classifier that will give the best classification performance. Also, it has been shown that the ensemble classifier performs better than direct techniques such as the greedy algorithm (Datta et al., 2010). For the greedy algorithm, the best performing candidate classifier is determined by using a combination of *k*-fold cross-validation and a weighted rank aggregation procedure. In this case, each candidate classifier in a user-defined set of classification algorithms is used to predict the hold-out set, and the performance measures utilized for evaluating the model are averaged across the *k* folds. Then, based on the resulting performance measures, a weighted rank aggregation procedure is used to rank the candidate classifiers, and the classifier with the top-most rank is chosen as the best performing classifier.

Furthermore, the ensemble classifier itself is an adaptive classification algorithm that excels in its flexibility in harnessing the strengths of the individual classifiers to yield better classification performance. However, we emphasize that an ensemble classifier will also depend on the set of candidate classifiers in its ensemble. To improve the performance of pre-selected candidate classifiers, the analyst may choose to tune hyperparameters of such classifiers since the default parameter settings in most software may not be ideal for the problem under consideration. In addition to adapting to the nature of the training data, techniques for handling class imbalance and dimension reduction can easily be incorporated into the construction of the ensemble classifier.

The apparent drawback of the ensemble classifier is the computational time required to train this classifier. The computing time is influenced by three main factors considered in the construction of the ensemble classifier - the number of candidate classifiers, the number of bootstrap samples for which each candidate classifier is trained, and the performance measures which are used in the weighted rank aggregation step of the construction procedure. Furthermore, the complexity of the candidate classifiers will also impact the computing times. For instance, more compute time will be required to train the MLP classifier than it will take to train the RPart classifier. If the analyst using the ensemble classifier chooses to tune the hyperparameters of the candidate classifiers, this will add some complexity to the process.

Nonetheless, the compute time can be reduced substantially by running the procedures described in section 2.6 in parallel on a computing cluster. For instance, consider the scenario where we utilized nine candidate classifiers (namely the candidate classifiers presented in Table 2), three performance measures for performing rank aggregation (namely Cohen's Kappa coefficient, G-mean, and MAUC), and 50 bootstrap samples. The construction of the ensemble classifier on a computing grid with an allocation of 10 CPU cores and a 120 GB memory limit takes an average of 9.47 h (wall-clock time). The supercomputer that we utilized for this operation has a total storage size of 2 petabytes, and 30,000 cores in Intel E5-2698v3 processors with 4 GB of RAM per core.

The classification methods described in this paper can help identify several microbial species linked to specific geographic locations. For instance, several species from genus *Bradyrhizobium* and *Pseudomonas* are amongst the top taxa responsible for differentiating between the city locations. This type of classification analysis can be a useful tool in identifying species that vary across different types of environments, climatic conditions, and surface types. The information from such analysis can be further synthesized in the fields such as forensics and disease epidemiology for identifying harmful pathogens.

While the identification of organisms is instrumental in metagenomics forensics, a good portion of sequenced reads remains unclassified when standard reference databases for taxonomic profiling. Sequenced reads which remain unclassified are likely to represent novel taxa, and ignoring such genetic data when constructing a fingerprint may impose substantial limitations. In this study, we utilized a *k*-mer approach for the direct modeling of DNA fragments obtained from metagenomics samples. For samples in the primary data set, classification results based on data generated from the *k*-mer approach were as good as the results from the analysis of taxonomic abundance data. However, the *k*-mer approach performs poorly in identifying the source origins of samples in the mystery set.

## Data Availability Statement

Publicly available datasets were analyzed in this study. This data can be found at: http://www.camda.info/.

## Author Contributions

SD and SG conceived and designed the study. SA-S and AS contributed equally in the analysis of the data and writing the draft. All authors contributed to editing the final manuscript.

## Conflict of Interest

The authors declare that the research was conducted in the absence of any commercial or financial relationships that could be construed as a potential conflict of interest.
