# Supplementary material for: Metagenomic Geolocation Prediction Using an Adaptive Ensemble Classifier
Source: Front Genet. 2021 Apr 20;12:642282. doi: 10.3389/fgene.2021.642282 (PMC8093763; doi:10.3389/fgene.2021.642282)
Supplement: Supplementary file 1 [file Data_Sheet_1.PDF]

Supplementary Material

1 SUPPLEMENTARY TABLES AND FIGURES

1.1 Tables

Table S1. The mean performance measures for a set of candidate classifiers and the ensemble classifier. Each classifier was trained on the primary data with a complete feature space. Three different scenarios are considered - the standard classifiers, classifiers trained with class weights, and classifiers trained with the implementation of an over-sampling procedure.

	Standard					Weighted					Over-Sampling				
Classifier	Accuracy	G-mean	Kappa	MAUC	Count	Accuracy	G-mean	Kappa	MAUC	Count	Accuracy	G-mean	Kappa	MAUC	Count
Ensemble	0.89	0.72	0.89	0.93	-	0.88	0.87	0.87	0.92	-	0.88	0.86	0.87	0.93	-
MLP	0.85	0.67	0.84	0.91	125	0.81	0.64	0.80	0.89	159	0.81	0.81	0.80	0.90	118
PCA+RF	0.09	0.00	0.03	0.64	0	0.10	0.00	0.04	0.66	0	0.08	0.00	0.03	0.66	0
PLS+RF	0.89	0.80	0.88	0.92	239	0.87	0.87	0.87	0.92	235	0.87	0.85	0.86	0.92	199
PLS+RPART	0.57	0.59	0.55	0.74	0	0.53	0.42	0.51	0.76	0	0.51	0.30	0.49	0.75	0
PLS+XGB	0.84	0.67	0.83	0.89	0	0.82	0.72	0.81	0.89	1	0.81	0.78	0.80	0.88	1
RF	0.85	0.51	0.85	0.91	18	0.85	0.51	0.84	0.92	47	0.82	0.63	0.81	0.91	38
RPART	0.56	0.57	0.54	0.76	0	0.58	0.58	0.56	0.79	1	0.56	0.45	0.54	0.78	0
SVM	0.77	0.67	0.76	0.87	1	0.10	0.10	0.00	0.50	0	0.74	0.63	0.72	0.86	0
XGB	0.89	0.80	0.88	0.93	117	0.87	0.85	0.86	0.91	57	0.85	0.83	0.84	0.91	144

Table S2. The mean performance measures for a set of candidate classifiers and the ensemble classifier. Each classifier was trained on the primary data with a feature reduced space. Three different scenarios are considered - the standard classifiers, classifiers trained with class weights, and classifiers trained with the implementation of an over-sampling procedure.

	Standard					Weighted					Over-Sampling				
Classifier	Accuracy	G-mean	Kappa	MAUC	Count	Accuracy	G-mean	Kappa	MAUC	Count	Accuracy	G-mean	Kappa	MAUC	Count
Ensemble	0.86	0.52	0.85	0.92	-	0.87	0.62	0.86	0.92	-	0.83	0.81	0.82	0.91	-
MLP	0.74	0.37	0.73	0.86	8	0.73	0.37	0.71	0.86	9	0.71	0.64	0.69	0.84	5
PCA+RF	0.10	0.00	0.04	0.65	0	0.11	0.00	0.04	0.64	0	0.11	0.00	0.06	0.67	0
PLS+RF	0.78	0.57	0.77	0.86	4	0.76	0.38	0.75	0.86	9	0.73	0.56	0.71	0.85	5
PLS+RPART	0.40	0.21	0.36	0.68	0	0.34	0.03	0.31	0.71	0	0.33	0.13	0.30	0.71	0
PLS+XGB	0.72	0.57	0.70	0.82	2	0.72	0.35	0.70	0.84	2	0.68	0.46	0.67	0.83	0
RF	0.86	0.35	0.85	0.92	260	0.87	0.62	0.86	0.92	372	0.84	0.81	0.83	0.91	290
RPART	0.56	0.47	0.53	0.76	1	0.57	0.39	0.55	0.79	0	0.54	0.36	0.51	0.76	0
SVM	0.41	0.39	0.36	0.69	0	0.10	0.10	0.00	0.50	0	0.45	0.48	0.41	0.72	0
XGB	0.86	0.76	0.85	0.92	225	0.85	0.84	0.84	0.90	108	0.83	0.81	0.82	0.90	200

Table S3. Total number of unique k-mers obtained from the samples in each of the 23 cities in primary dataset and the samples in the mystery set.

Location Code	Collection	Sample size	unique k-mers
ARN	CSD17	50	1,910,929
BCN	CSD16	38	28,047,151
BER	CSD16	41	56,282,452
DEN	CSD16	23	3,381,633
	CSD17	22	3,240,933
DOH	CSD16	50	1,507,989
	CSD17	15	4,955,758
FAI	CSD16	48	124,038,615
HKG	CSD17	49	47,288,326
ICN	CSD17	50	3,288,036
IEV	CSD17	49	22,845,501
ILR	CSD16	47	411,558,550
	CSD17	50	19,821,131
KUL	CSD17	30	17,911,255
LCY	CSD17	37	64,328,000
LIS	CSD16	19	131,462,884
NYC	CSD16	49	173,020,781
	CSD17	50	22,507,397
OFF	CSD16	26	268,185,127
SAO	CSD17	29	4,969,518
SCL	CSD16	26	22,018,936
SDJ	CSD17	32	1,628,278
SFO	CSD17	29	670,649
SGP	CSD17	48	17,253,003
TPE	CSD17	50	33,687,013
TYO	CSD16	25	45,854,587
	CSD17	50	11,085,782
ZRH	CSD17	33	39,091,295
Mystery	-	121	17,327,339
